# Editorial: Dietary pattern and metabolic syndrome

**DOI:** 10.3389/fnut.2026.1774774

**Published:** 2026-02-06

**Authors:** Shaojie Liu, Yingnan Jia, Mengyun Luo

**Affiliations:** 1 Department of Clinical Nutrition, School of Medicine, The First Affiliated Hospital of Xiamen University, Xiamen University, Xiamen, China; 2 Key Lab of Public Health Safety of the Ministry of Education, School of Public Health, Fudan University, Shanghai, China; 3 Prevention Research Collaboration, Sydney School of Public Health, The University of Sydney, Sydney, NSW, Australia

**Keywords:** butyrate, dietary patterns, foods, functional food components, functional nutrients, ketogenic, macro level dietary patterns, metabolic syndrome

## Introduction

Metabolic Syndrome (MetS), a clustering of obesity, hypertension, dysglycemia, and dyslipidemia, is a major global driver of type 2 diabetes and cardiovascular disease. Its rising prevalence represents a formidable public health challenge. While diet is a cornerstone of MetS management, the effectiveness of general recommendations is limited by profound variations in food consumption and dietary patterns across different age groups and regions. This Research Topic addresses this gap by focusing on the diet-MetS relationship across the lifespan and diverse geographies, with special attention to dietary assessment in different age groups. Our goal is to identify key, context-specific dietary patterns to inform more dietray suggestions of MetS.

## Topic overview: building an evidence chain from functional nutrients/foods to macro-patterns

The 13 studies included in this Research Topic can be broadly categorized into two groups based on their focus—either on functional nutrients/foods or on overall dietary patterns. Together, they complement each other and advance our understanding of the “diet-metabolism” relationship.

## Category 1: the metabolic regulatory role of functional nutrients and foods

This group of studies delves into the direct effects of specific food components or functional nutrients on MetS components, offering candidate solutions for targeted nutritional interventions.

- Pan et al. find that yacon root improves bowel habits and reduces body weight, waist circumference, postprandial blood glucose, and triglyceride levels, supporting its potential as a functional food for weight management and constipation relief.
- Chen et al. reveal that the SOD1 gene polymorphism (rs2070424) can modify the associations of plasma zinc/copper levels (and their ratio) with renal function. This highlights the complex role of gene-nutrients interactions in metabolic health.
- Zhao, Zhao, Li et al. focus on gut metabolites. They find that dietary and circulating butyrate levels are independently associated with better renal function (eGFR) in diabetic patients, offering a new perspective on how dietary fiber influences renal health via gut microbiota metabolism.
- Hidayat et al. confirm the protective effect of soy intake in a Chinese adult population. Each additional 25 grams per day of soy intake was associated with a significantly reduced risk of MetS and improvement of its components (waist circumference, triglycerides, high blood pressure, etc.).
- Fu et al. evaluate the metabolic benefits of spirulina supplementation as an adjuvant therapy in overweight/obese individuals, including improvements in blood lipids, blood pressure, and body weight. They also suggest that combining spirulina with exercise may yield enhanced effects.
- Bai et al. build an obesity risk prediction model to identify that a “Vegetable-Meat- Grain” and “Milk-Egg” dietary patterns are associated with a lower risk of high body fat percentage, while “Snack” and “Aquatic-Meat” patterns increase the risk.

## Category 2: epidemiological and intervention studies on dietary patterns and metabolic health

This group of studies examines the impact of overall dietary structures on MetS and related disease risks, encompassing short-term interventions, analyses of regional patterns, and risk assessment tools.

- Zhou et al. demonstrate that short-term very-low-calorie diets can effectively improve body weight, waist circumference, BMI, blood pressure, and glucose/lipid metabolism markers in MetS patients, providing evidence for an intensive short-term clinical management strategy.
- Basnet et al. identify a dietary pattern characterized by high protein intake, fried foods, sweets, and desserts on a population of southeastern coastal China increases the risk of hyperuricemia, underscoring the hazards of region-specific unhealthy eating patterns.
- Liu et al. reveal specific associations between dietary patterns and obesity phenotypes in a study of adults in Inner Mongolia. A “Northern Pastoral” dietary pattern increases the risk of metabolically healthy obesity (MHO), while a “Plant-based” pattern reduces it, indicating that dietary impacts must be considered in conjunction with metabolic phenotypes.
- Chen and Yao, in a comprehensive review, systematically elaborate on the potential mechanisms, practical strategies, and future directions of ketogenic diets in managing MetS, providing a thorough reference for the application and critical evaluation of this extreme carbohydrate-restriction model.
- Zhao, Zhao, Fu et al. find that a higher score of Oxidative Balance Score (OBS) is associated with a reduced risk of chronic kidney disease. This serves as an example of using comprehensive scoring tools for risk prediction.
- Lu et al. reveal that low HDL-C might be a protective factor against instrumental activities of daily living (IADL) disability, while the combination of abdominal obesity, elevated blood pressure, and high fasting blood glucose poses the greatest threat to activities of daily living and IADL in the elderly in a multi-ethnic region of southwestern China.
- Qiao et al. demonstrate a significant, negative, and non-linear association between the Geriatric Nutritional Risk Index (GNRI) and all-cause as well as cardiovascular mortality in U.S. diabetic population, highlighting the importance of comprehensive nutritional status for long-term prognosis.

## Synthesis and future directions

Synthesizing the findings from this Research Topic, several key insights emerge. First, the influence of diet on MetS operates across all levels, from specific functional components (e.g., butyrate, trace elements) to macro dietary patterns (e.g., plant-based, ketogenic), necessitating a multi-layered, integrated consideration. Second, the diet-MetS relationship is significantly modified by age (e.g., university students, the elderly), region (e.g., southeastern coast, Inner Mongolia, southwestern multi-ethnic region), genetic background (e.g., SOD1 genotype), and metabolic phenotype (e.g., MHO).

The research in this Research Topic also reveals areas of consensus and directions for further exploration. A consensus exists that increasing the intake of plant-based foods and functional foods (e.g., soy, yacon root), improving overall diet quality (high OBS), and enhancing nutritional status (high GNRI) have protective effects on metabolic health, either universally or in specific subpopulations. Conversely, energy-dense, nutrient-poor dietary patterns (e.g., high in snacks, fried foods, and sweets) generally increase risk.

Future research must prioritize: (1) Longitudinal and intervention studies in specific age groups (children, elderly) to define life-stage-appropriate dietary patterns; (2) Integrated multi-omics studies to decipher the biological mechanism of dietary pattern on the metabolic diseases; (3) Leveraging digital tools and machine learning to integrate dietary, clinical, and genetic data for dynamic risk prediction and management.

## Conclusion

The studies in this Research Topic collectively illustrate that combating MetS requires dietary strategies calibrated to population and individual heterogeneity. From functional nutrients/foods to macro dietary patterns, each article contributes a vital piece to the precision nutrition interference for metabolic diseases. We hope this work stimulates researchers and practitioners to develop and implement more nuanced, effective, and personalized dietary patterns to mitigate the global burden of metabolic diseases.

